# Ultra-high voltage electron microscopy of primitive algae illuminates 3D ultrastructures of the first photosynthetic eukaryote

**DOI:** 10.1038/srep14735

**Published:** 2015-10-06

**Authors:** Toshiyuki Takahashi, Tomoki Nishida, Chieko Saito, Hidehiro Yasuda, Hisayoshi Nozaki

**Affiliations:** 1 Department of Biological Sciences, Graduate School of Science, University of Tokyo, 7-3-1 Hongo, Bunkyo-ku, Tokyo, 113-0033, Japan; 2 Research Center for Ultra-High Voltage Electron Microscopy, Osaka University, 7-1 Mihogaoka, Ibaraki, Osaka 567-0047, Japan

## Abstract

A heterotrophic organism 1–2 billion years ago enslaved a cyanobacterium to become the first photosynthetic eukaryote, and has diverged globally. The primary phototrophs, glaucophytes, are thought to retain ancestral features of the first photosynthetic eukaryote, but examining the protoplast ultrastructure has previously been problematic in the coccoid glaucophyte *Glaucocystis* due to its thick cell wall. Here, we examined the three-dimensional (3D) ultrastructure in two divergent species of *Glaucocystis* using ultra-high voltage electron microscopy. Three-dimensional modelling of *Glaucocystis* cells using electron tomography clearly showed that numerous, leaflet-like flattened vesicles are distributed throughout the protoplast periphery just underneath a single-layered plasma membrane. This 3D feature is essentially identical to that of another glaucophyte genus *Cyanophora*, as well as the secondary phototrophs in Alveolata. Thus, the common ancestor of glaucophytes and/or the first photosynthetic eukaryote may have shown similar 3D structures.

Approximately 1–2 billion years ago during the Proterozoic Eon, a heterotrophic eukaryote enslaved a cyanobacterium to obtain the ability for photosynthesis and become the common ancestor of the primary photosynthetic eukaryotes [Archaeplastida^1^,^2^ or Kingdom Plantae *sensu* Cavalier-Smith (1981)^3^,^4^]. Primary photosynthetic eukaryotes have ruled this planet as primary producers, evolving into species of three major lineages^1^,^2^,^5^,^6^; namely, red algae thriving throughout the ocean, Chloroplastida [Viridiplantae (green algae and land plants)] advancing onto land, and glaucophytes (Fig. 1 and Supplementary Fig. 1). The glaucophytes comprise the smallest number of taxa among these three lineages, and are rare microalgae that live only in inland freshwater^7^,^8^,^9^. Furthermore, the glaucophytes are considered enigmatic phototrophs retaining the ancestral archaeplastidal features (represented by the peptidoglycan wall surrounding the plastids) that may have been lost in red algae and Chloroplastida^5^,^9^,^10^,^11^. For this reason, the flagellate glaucophyte species *Cyanophora paradoxa* (Supplementary Fig. 1) has been widely examined as a model organism to resolve the most ancestral features of photosynthetic eukaryotes^6^,^12^,^13^,^14^,^15^,^16^,^17^.

Cavalier-Smith^18^ considered that the glaucophytes, dinophytes (Alveolata^1^) and Euglenozoa (Excavata^1^) might be quite closely related because of the presence of alveolate pellicle (protoplast periphery with “flattened vesicles” and/or plates distributed just underneath the plasma membrane) in these three groups. He recently hypothesised that “cortical alveoli” (flattened vesicles) may have evolved in the common ancestor of a large eukaryotic group, “corticates” composed of primary phototrophs (Plantae or Archaeplastida) and Chromista (including Chromalveolata^1^ and Rhizaria^1^)^19^. Spiegel^5^ discussed that the first photosynthetic eukaryote may have been a *Cyanophora*-like flagellate. As supported by ultrathin section transmission electron microscopy (TEM) and freeze-fracture TEM^20^,^21^,^22^, field emission scanning electron microscopy (FE-SEM) recently showed that the whole peripheral surface of naked vegetative cells in several species of *Cyanophora* is ornamented with angular fenestrations formed by ridges structured by overlapping, leaflet-like flattened vesicles underneath the plasma membrane^21^,^22^. However, this leaflet-like 3D morphology of the flattened vesicles has not been unambiguously demonstrated in other glaucophyte genera, possibly because FE-SEM cannot reveal surface ultrastructures of the periphery of the protoplast that is enclosed by a cell wall or extracellular matrix in these genera^7^. Although freeze-fracture TEM revealed leaflet-like surface appearances of flattened vesicles in the coccoid glaucophyte genus *Glaucocystis*^23^,^24^, the 3D ultrastructural features of the *Glaucocystis* protoplast periphery are unclear, especially regarding the spatial relationship between the plasma membrane and flattened vesicles^23^,^24^,^25^,^26^. *Cyanophora* represents one of the two divergent clades of glaucophytes; the other clade includes *Cyanoptyche, Gloeochaete*, and *Glaucocystis*^27^. Thus, to provide more detailed ancestral features of glaucophyte cells, ultrastructural characterisation of 3D structures of the protoplast periphery in the latter three glaucophyte genera is required.

Recent advancements in ultra-high voltage electron microscopy (UHVEM) have enabled thick-section micrographs in biological samples^28^. Based on 3D UHVEM tomography, the *in situ* peripheral ultrastructure of protoplasts can be observed, even when enclosed by extracellular structures^29^. However, 3D UHVEM has not previously been applied to algae or protozoa.

To examine the peripheral 3D ultrastructure of *Glaucocystis* protoplasts enclosed by a cell wall, we performed 3D-modelling based on the UHVEM tomography using high-pressure freezing (HPF) and freeze-substitution (FS) fixation of two divergent strains of *Glaucocystis*: “*G. geitleri*” SAG 229-1 and *G. nostochinearum* SAG 16.98^22^,^27^ (Fig. 1).

## Results

**Figure 2:**
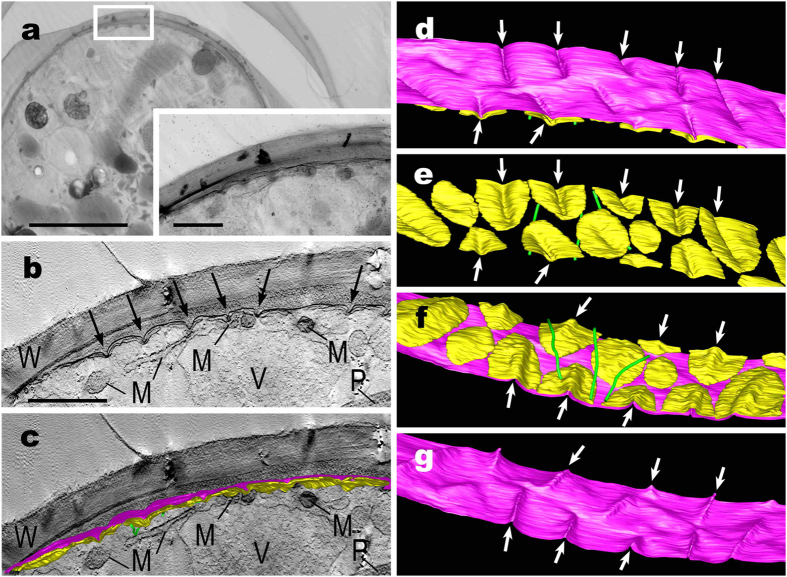
Electron tomography and 3D-modelling of protoplast periphery of “*Glaucocystis geitleri*” SAG 229-1 vegetative cell. Corresponding to Supplementary Movies 1 and 2. (**a**) Ultra-high voltage electron microscopic image. Inset shows higher magnification image of the cell periphery (boxed area). Scale bar, 5 μm and 1 μm (inset). (**b**) Tomographic image of boxed area in (**a**). Note that plasma membrane is grooved deeply at regular intervals (arrows). Scale bar, 1 μm. M, mitochondrion; P, plastid; V, vacuole; W, cell wall. (**c–g**) 3D images showing distribution of plasma membrane (magenta), and underlying flattened vesicles (yellow) associated with microtubules (green) on cytoplasmic side. Not to scale. Arrows indicate that each bar-like groove of plasma membrane is covered by invagination of flattened vesicle. (**c**) View with a tomographic image. For abbreviations of organelles, see (**b**). (**d,e**) View from cell wall side. (**f,g**) View from cytoplasmic side.

**Figure 3:**
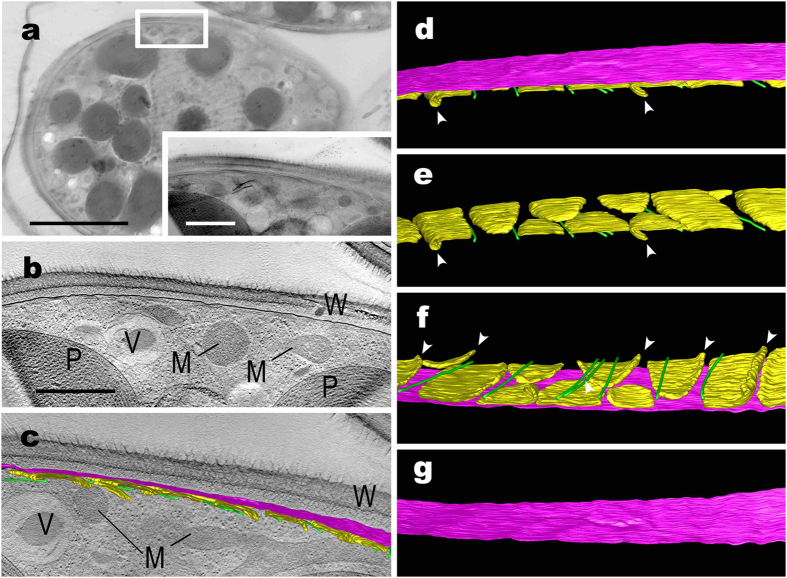
Electron tomography and 3D-modelling of protoplast periphery of *Glaucocystis nostochinearum* SAG 16.98 vegetative cell. Corresponding to Supplementary Movies 3 and 4. (**a**) Ultra-high voltage electron microscopic image. Inset shows higher magnification image of the cell periphery (boxed area). Scale bar, 5 μm and 1 μm (inset). (**b**) Tomographic image of boxed area in (**a**). Note that the plasma membrane lacks deep grooves at section. Scale bar, 1 μm. M, mitochondrion; P, plastid; V, vacuole; W, cell wall. (**c–g**) 3D images showing distribution of plasma membrane (magenta), and underlying flattened vesicles (yellow) associated with microtubules (green) on cytoplasmic side. Not to scale. Note that plasma membrane and flattened vesicles exhibit almost smooth surfaces. Arrowheads indicate slight overlapping of neighbouring flattened vesicles. (**c**) View with a tomographic image. For abbreviations of organelles, see (**b**). (**d,e**) View from the cell wall side. (**f,g**) View from the cytoplasmic side.

Using UHVEM tomography, the 3D ultrastructural features of the plasma membrane and the flattened vesicles at the protoplast periphery of the two *Glaucocystis* species were visualised with high contrast (Figs 2 and 3 and Supplementary Movies 1,2,3,4). In both species, the flattened vesicles were leaflet-like in shape, lacked a plate-like interior structure, and were distributed throughout the entire protoplast periphery just underneath the single-layered plasma membrane (except for the region near basal bodies; see below), but did not completely enclose the protoplast periphery to form small spaces between the vesicles at the protoplast periphery.

However, our comparative 3D-modelling based on the peripheral tomography clearly showed essential differences in the protoplast periphery between the two species (Figs 2 and 3 and Supplementary Movies 2 and 4). We observed various regions of matured vegetative cells by UHVEM and tomography, as well as ultrathin section TEM (Supplementary Fig. 2 and Supplementary Note 1); the peripheral 3D structures were essentially consistent within each species. In “*G. geitleri*” SAG 229-1 cells (Fig. 2 and Supplementary Movies 1 and 2), the plasma membrane exhibited bar-like grooves when viewed from the outside (or bar-like ridges when viewed from the inside) (Fig. 2b–d,g). These grooves were measured to be 500–1,500 nm long, 60–90 nm wide, and 100–150 nm deep; they were arranged almost in parallel at regular intervals of 500–800 nm. The flattened vesicles just below the plasma membrane were 30–70 nm thick and almost ellipsoidal or ovoid in front view (700–2,000 nm long and 300–600 nm wide) with a bar-like invagination in the centre when viewed from the outside (Fig. 2b,c,e). The invagination of the flattened vesicle was measured to be 500–1,500 nm long, 80–110 nm wide, and 100–150 nm deep. Each groove on the plasma membrane was backed almost entirely with the invagination of the flattened vesicle just underneath the plasma membrane; the backing was often associated with microtubules arranged in parallel (Fig. 2f). The flattened vesicles were almost separated from one another at the protoplast periphery of “*G. geitleri*” SAG 229-1 cells. Many elongated mitochondria were observed below the flattened vesicles at the protoplast periphery (Fig. 2b).

In *G. nostochinearum* SAG 16.98 cells (Fig. 3 and Supplementary Movies 3 and 4), the plasma membrane was almost flat in surface view (Fig. 3b–d,g), lacking the depression or invagination observed in “*G. geitleri*” SAG 229-1. The flattened vesicles just underneath the plasma membrane neighboured the inner surface of the plasma membrane at regular patterns in *G. nostochinearum* SAG 16.98 (Fig. 3b,c,e,f). The vesicles were 30–70 nm thick and elongate-cylindrical in front view (1,500–2,000 nm long and 500–1,000 nm wide); they were almost smooth from a surface view (Fig. 3e). Their marginal regions were often slightly overlapped with one another (Fig. 3f).

**Figure 4:**
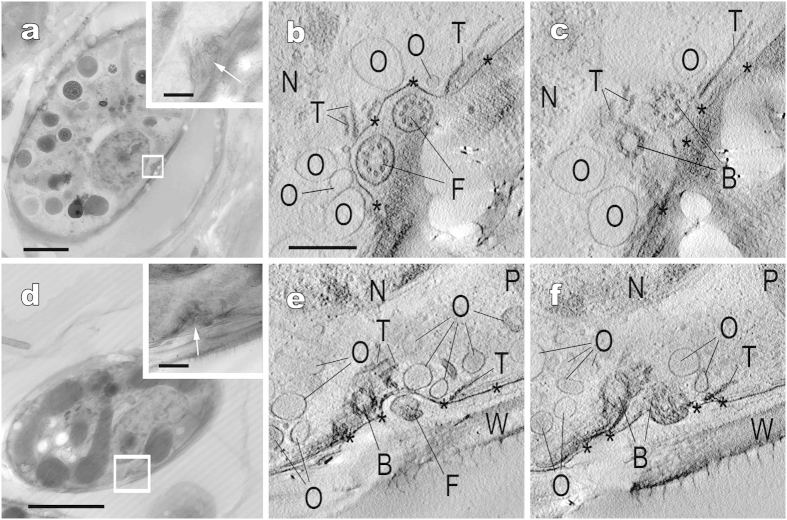
Electron tomography of the cell periphery near basal bodies of “*Glaucocystis geitleri*” SAG 229-1 (**a–c**) and *G. nostochinearum* SAG 16.98 (**d–f**). Corresponding to **Supplementary Movies 5 and 6.** (**a**,**d**) Ultra-high voltage electron microscopic images of vegetative cells. Insets show higher magnification images in boxed area. Scale bar, 5 μm and 500 nm (insets). (**b**,**c**,**e**,**f**) Tomographic images of boxed area in (**a**,**d**), showing portions of cell periphery near basal bodies and vestigial flagella. Shown at the same magnification. Note that the cell periphery in these areas is composed of plasma membrane (asterisks) and ovoid-to-spherical vesicles surrounding basal bodies. Scale bar, 500 nm. B, basal body; F, vestigial flagellum; N, nucleus; O, ovoid-to-spherical vesicle; P, plastid; T, microtubule; W, cell wall.

Although vestigial flagella in *Glaucocystis* cells have previously been observed by ultrathin section TEM^23^,^24^,^25^,^26^, our UHVEM tomography clearly showed the 3D ultrastructure of the protoplast periphery surrounding basal bodies and neighbouring vestigial flagella in “*G. geitleri*” SAG 229-1 (Fig. 4a–c and Supplementary Movie 5) and *G. nostochinearum* SAG 16.98 (Fig. 4d–f and Supplementary Movie 6). The 3D structure of the protoplast periphery surrounding basal bodies and neighbouring vestigial flagella in the two species was essentially identical. Two vestigial flagella were situated between the cell wall and protoplast periphery at the cell equator and positioned within the furrow of the protoplast surface, and were connected to the basal bodies within the cytoplasm. Flattened vesicles were lacking near the basal bodies and flagella as observed previously^24^ but the present UHVEM clearly revealed ovoid-to-spherical vesicles distributed below the plasma membrane near the basal bodies and flagella (Fig. 4 and Supplementary Movies 5 and 6).

## Discussion

The present UHVEM tomography study clearly demonstrated that the plasma membrane of “*G. geitleri*” SAG 229-1 represented a single, continuous sheet with numerous bar-like grooves that were distributed throughout the surface; the grooves were associated with numerous, leaflet-like flattened vesicles just underneath the plasma membrane (Fig. 2). Except for the presence of grooves, these ultrastructural features of the protoplast periphery in “*G. geitleri*” SAG 229-1 were essentially the same as those of *G. nostochinearum* SAG 16.98 (Fig. 3), as well as five species of the motile glaucophyte genus *Cyanophora*^21^,^22^; a single plasma membrane is closely associated with numerous, leaflet-like flattened vesicles distributed throughout the periphery just underneath the membrane. Thus, these 3D structures can be considered common ancestral features of the glaucophytes. Even when 3D structures had not been clarified and molecular data were lacking, Kies^26^ already considered the peripheral flattened vesicles (“Lakunensystem”) as a unifying morphological characteristic of glaucophytes.

**Figure 5:**
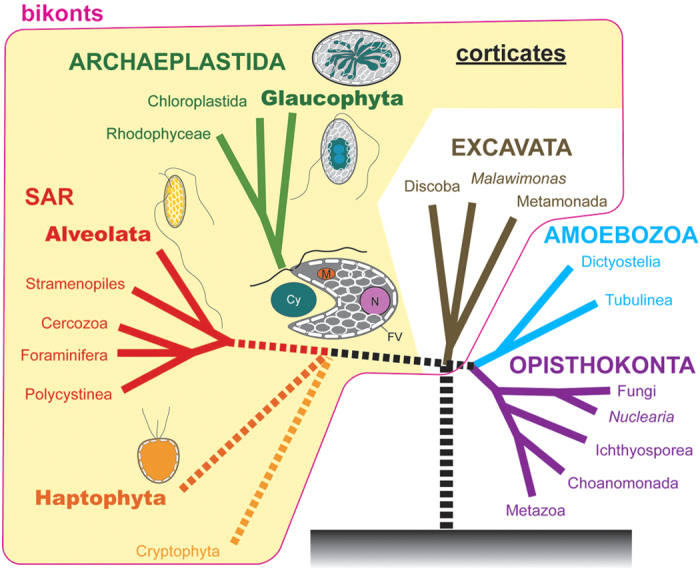
Diagram of a possible evolutionary scenario from an ancestral flagellate with leaflet-like flattened vesicles to extant primary photosynthetic eukaryotes (Archaeplastida). Based on the present study, Adl *et al.*^2^ and Cavalier-Smith^19^. Extant organisms that possibly retain flattened vesicles are Glaucophyta, Alveolata and Haptophyta. The putative ancestral flagellate of glaucophytes or the first primary photosynthetic eukaryote enslaved a cyanobacterium (Cy) as plastids might have contained nucleus (N), mitochondria (M) and leaflet-like flattened vesicles (FV).

In dinophytes and *Chromera* (Alveolata), similar 3D structures of the plasma membrane and the underlying leaflet-like flattened vesicles or alveolae can be considered based on SEM/FE-SEM and ultrathin section TEM^2^,^30^,^31^,^32^. In addition, some haptophytes possess flattened-vesicle-like ultrastructures or peripheral endoplasmic reticulum (PER) just beneath the plasma membrane^33^. Thus, fundamentally identical or homologous peripheral ultrastructures may be distributed in separate lineages or different supergoups within corticates or bikonts (corticates plus Excavata, or eukaryotes excluding Amoebozoa and Opisthokonta^2^) (Fig. 5). On the other hand, no organism in the other two groups of Archaeplastida (Chloroplastida and red algae) and unikonts (composed of opisthokonts and amoebozoans) contains such complicated peripheral ultrastructures. Given that the glaucophytes represent the most ancestral features of Archaeplastida^5^,^6^, the leaflet-like flattened vesicles in the protoplast periphery in glaucophyte cells may have been retained from the first photosynthetic eukaryote in the Precambrian period or a more ancient ancestor within the bikonts, as suggested by Cavalier-Smith^19^ (Fig. 5). In the ancestors of Chloroplastida and red algae, the flattened vesicles may have been lost during evolution.

The flattened vesicles of *Cyanophora* contain a plate and completely enclose the protoplast by overlapping with one another at the protoplast periphery to form ridges on the cell surface under FE-SEM^21^,^22^. In contrast, the present study demonstrated that two divergent species of *Glaucocystis* have flattened vesicles that lack the plate and are more or less separated from one another just underneath the plasma membrane to form spaces between the vesicles at the protoplast periphery. This difference may reflect the presence or absence of a cell wall in these two genera. Since the *Cyanophora* cells lack cell walls, the function of tightly arranged flattened vesicles with plates may protect the protoplast or facilitate the formation of cell shape characteristics of the species^21^. It is generally believed that the flagellate vegetative cells represent an ancestral form in the photosynthetic eukaryotes or algae^5^,^34^. Thus, it is possible that during evolutionary processes from the ancestral *Cyanophora*-like flagellate to the immotile *Glaucocystis* cell, the flattened vesicles in the protoplast periphery may have lost their plates in exchange for obtaining a wall to protect the cell periphery.

In lacking motile stages during life cycle in cultured material^23^, the vestigial flagella of *Glaucocystis* vegetative cells can be considered to be a non-functional organ or evolutionary remnant of flagella of the ancient flagellate ancestor. Thus, *Glaucocystis* may represent early evolutionary stage from flagellate vegetative cells to nonmotile vegetative cells. The present study clearly showed that flattened vesicles are lacking but ovoid-to-spherical vesicles are distributed below the plasma membrane near the basal bodies and vestigial flagella (Fig. 4 and Supplementary Movies 5 and 6). Given that these two types of vesicles have different functions, the vestigial flagella may have a cryptic function (e. g. sensory organelle as in neural cilia^35^,^36^,^37^) in communication with the surrounding cytoplasmic periphery that harbours the ovoid-to-spherical vesicles.

Based on morphological comparison at the species level, ultrastructural differences were resolved between the two divergent *Glaucocystis* species in the plasma membrane and the underlying flattened vesicles (Figs 2 and 3 and Supplementary Movies 2 and 4). In “*G. geitleri*” SAG 229-1, the plasma membrane and the underlying flattened vesicles formed numerous bar-like grooves that were distributed throughout the protoplast surface, and the flattened vesicles were almost separated from one another at the protoplast periphery (Fig. 2 and Supplementary Movie 2). In contrast, the plasma membrane and vesicles of *G. nostochinearum* SAG 16.98 were almost smooth or flat, lacking such grooves or invaginations, and the vesicles were often slightly overlapping with one another at the periphery of the protoplast (Fig. 3 and Supplementary Movie 4). Thus, ultrastructural diversity of the protoplast periphery is apparent within the genus *Glaucocystis*, in contrast to previous reports^25^,^38^.

## Conclusions

In the present study, the 3D ultrastructural arrangement of the plasma membrane and the underlying leaflet-like flattened vesicles in the coccoid glaucophyte genus *Glaucocystis* were clearly observed by UHVEM tomography and 3D-modelling using HPF-FS method. Although plates are lacking within the vesicles, the *Glaucocystis* periphery is essentially identical based on the 3D ultrastructural arrangement as that of motile glaucophyte genus *Cyanophora*, as well as alveolates, which suggests that such peripheral ultrastructures may represent ancestral features of the first photosynthetic eukaryote, as well as the first corticate, as suggested by Cavalier-Smith^19^. At the species level, the two species of *Glaucocystis* were clearly distinguished from each other based on our ultrathin section TEM as well as 3D UHVEM tomographic comparison of peripheral ultrastructures just inside the wall. Hence, UHVEM tomography can be used to explore the 3D ultrastructural arrangement of the periphery, even in the presence of a wall or extracellular matrix, and can be used to compare the subcellular ultrastructure or 3D arrangement of organisms. Thus, further 3D UHVEM tomography and 3D-modelling of other strains or species of *Glaucocystis*, as well as for other bikonts, will unveil the actual diversity and ancestral ultrastructural features of the bikonts.

## Methods

### Strains and culture conditions

“*Glaucocystis geitleri*” SAG 229-1 and *G. nostochinearum* SAG 16.98 were obtained from the Sammlung von Algenkulturen der Universität Göttingen^39^ (SAG, http://sagdb.uni-goettingen.de/). SAG 229-1 is one of the most widely used *Glaucocystis* strains and originates from Pringsheim’s “authentic” strain of “*Glaucocystis geitleri* nom. provis”. (provisional name by Pringsheim^40^ without species description)^41^. SAG 16.98 is labelled as the type species “*G. nostochinearum*” and was collected in Germany^39^ (http://sagdb.uni-goettingen.de/) where the type locality of *G. nostochinearum* is located^42^. The cultures were maintained in screw-cap tubes with 9–11 mL AAF-6 medium^43^,^44^ under 14 h-light/10 h-dark conditions at 20 °C with a photon flux density of *ca*. 50–60 μmol/m^2^/s.

### High-pressure freezing (HPF) and freeze-substitution (FS) fixation

Since the HPF-FS fixation method is generally expected to be superior to chemical fixation in preserving the integrity of cellular ultrastructure^13^,^45^,^46^, this method was performed for TEM and UHVEM as described previously^13^,^46^ with minor modifications. Briefly, cells were harvested directly from the cultures using a pipette and frozen under high pressure using a high-pressure freezing machine (HPM010; Bal-Tec). The samples were placed onto frozen 4% osmium tetroxide anhydrous acetone at liquid-nitrogen temperature and post-fixed in the solution incubated at −80 °C for 5 days before warming gradually to −20 °C for 2 h, then to 4 °C for 1 h and finally to room temperature. The samples were washed three times with anhydrous acetone and infiltrated with increasing concentrations of Spurr’s resin^47^ in anhydrous acetone, and finally embedded in Spurr’s resin.

### Transmission electron microscopy (TEM) and ultra-high-voltage electron microscopy (UHVEM)

Ultrathin section TEM was performed as described previously^48^. Prior to UHVEM observation, thick sections (1 or 2 μm) were cut using an ultramicrotome (Ultracut E, Reichert-Jung) and mounted on formvar-coated copper grids. The thick sections were stained in 10% uranyl acetate in 70% methanol with 150 W microwave for 30 s and then incubated for 20 min. After washing and drying, the sections were stained in lead citrate with 150 W microwave for 30 s and then incubated for 10 min. Colloidal gold particles (20 or 60 nm in diameter) were deposited on both sides of each section, and the samples were observed using UHVEM (H-3000, Hitachi) at an accelerating voltage of 2 MV. Tomographic image series were recorded using a 4096 × 4096 pixel slow scan CCD camera (TVIPS). Single axis tilt series were obtained from ±60° with 2° increments. Reconstruction of the tomographic and 3D-modelling was performed as described previously^29^.

## Additional Information

**How to cite this article**: Takahashi, T. *et al.* Ultra-high voltage electron microscopy of primitive algae illuminates 3D ultrastructures of the first photosynthetic eukaryote. *Sci. Rep.* **5**, 14735; doi: 10.1038/srep14735 (2015).

## Supplementary Material

Supplementary Information

Supplementary Movie 1

Supplementary Movie 2

Supplementary Movie 3

Supplementary Movie 4

Supplementary Movie 5

Supplementary Movie 6

## Figures and Tables

**Figure 1:**
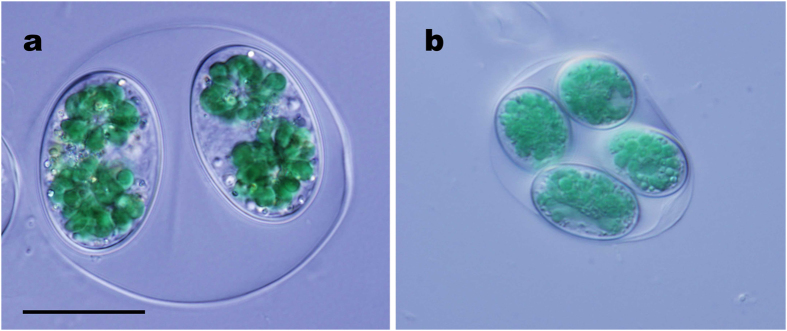
Differential interference contrast microscopy of vegetative cells of two species of the coccoid glaucophyte genus *Glaucocystis*. Shown at the same magnification. Scale bar, 20 μm. Note that immobile vegetative cells are enclosed by a cell wall within an expanded mother cell wall. (**a**) “*G. geitleri*” SAG 229-1. (**b**) *G. nostochinearum* SAG 16.98.
