# Supplementary material for: Ultra-high voltage electron microscopy of primitive algae illuminates 3D ultrastructures of the first photosynthetic eukaryote
Source: Sci Rep. 2015 Oct 6;5:14735. doi: 10.1038/srep14735 (PMC4593968; doi:10.1038/srep14735)
Supplement: Supplementary Information [file srep14735-s1.doc]

**Supplementary Information**

**Ultra-high voltage electron microscopy of primitive algae illuminates 3D ultrastructures of the first photosynthetic eukaryote**

Toshiyuki Takahashi, Tomoki Nishida, Chieko Saito, Hidehiro Yasuda & Hisayoshi Nozaki^*^

**Affiliations**

Department of Biological Sciences, Graduate School of Science, University of Tokyo, 7-3-1 Hongo, Bunkyo-ku, Tokyo, 113-0033, Japan

Toshiyuki Takahashi, Chieko Saito & Hisayoshi Nozaki^*^

Research Center for Ultra-High Voltage Electron Microscopy, Osaka University, 7-1 Mihogaoka, Ibaraki, Osaka 567-0047, Japan

Tomoki Nishida & Hidehiro Yasuda

**Contact information**

Correspondence to: Hisayoshi Nozaki^*^

e-mail: nozaki@bs.s.u-tokyo.ac.jp

**
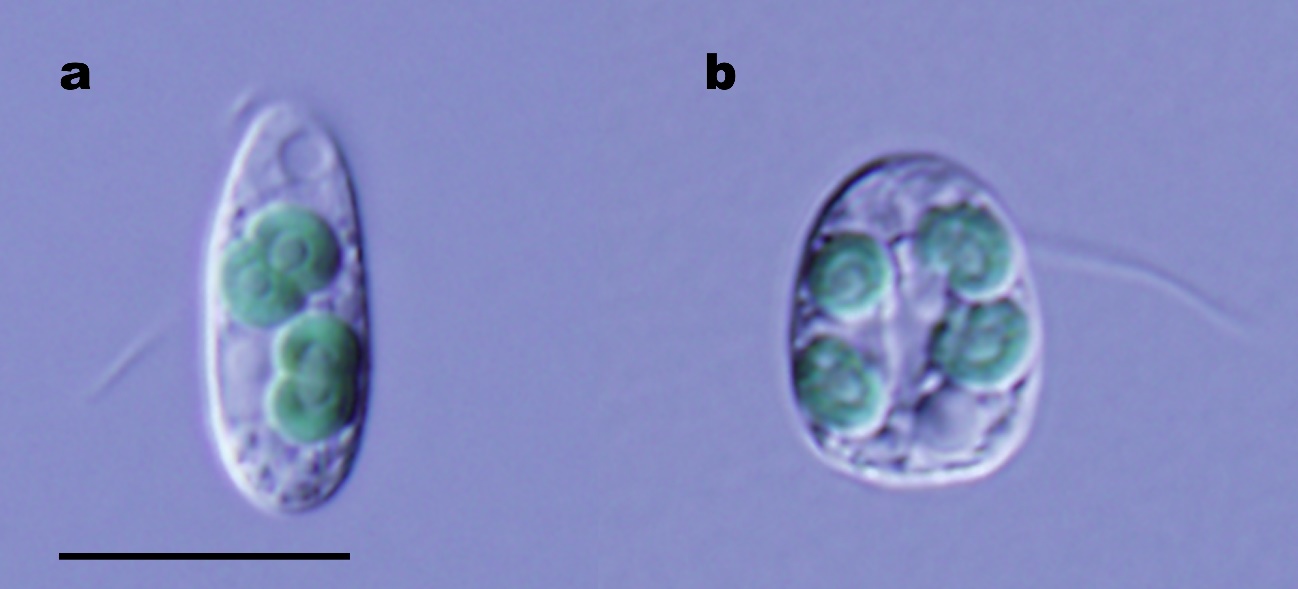
**

**Supplementary Figure 1 | Differential interference contrast microscopy of two species of vegetative cells of the motile glaucophyte genus *Cyanophora****.* Shown at the same magnification. Note that the flagellate vegetative cells are naked. Scale bar, 10 µm. (**a**) *C.* *paradoxa*. (**b**) *C.* *sudae.*


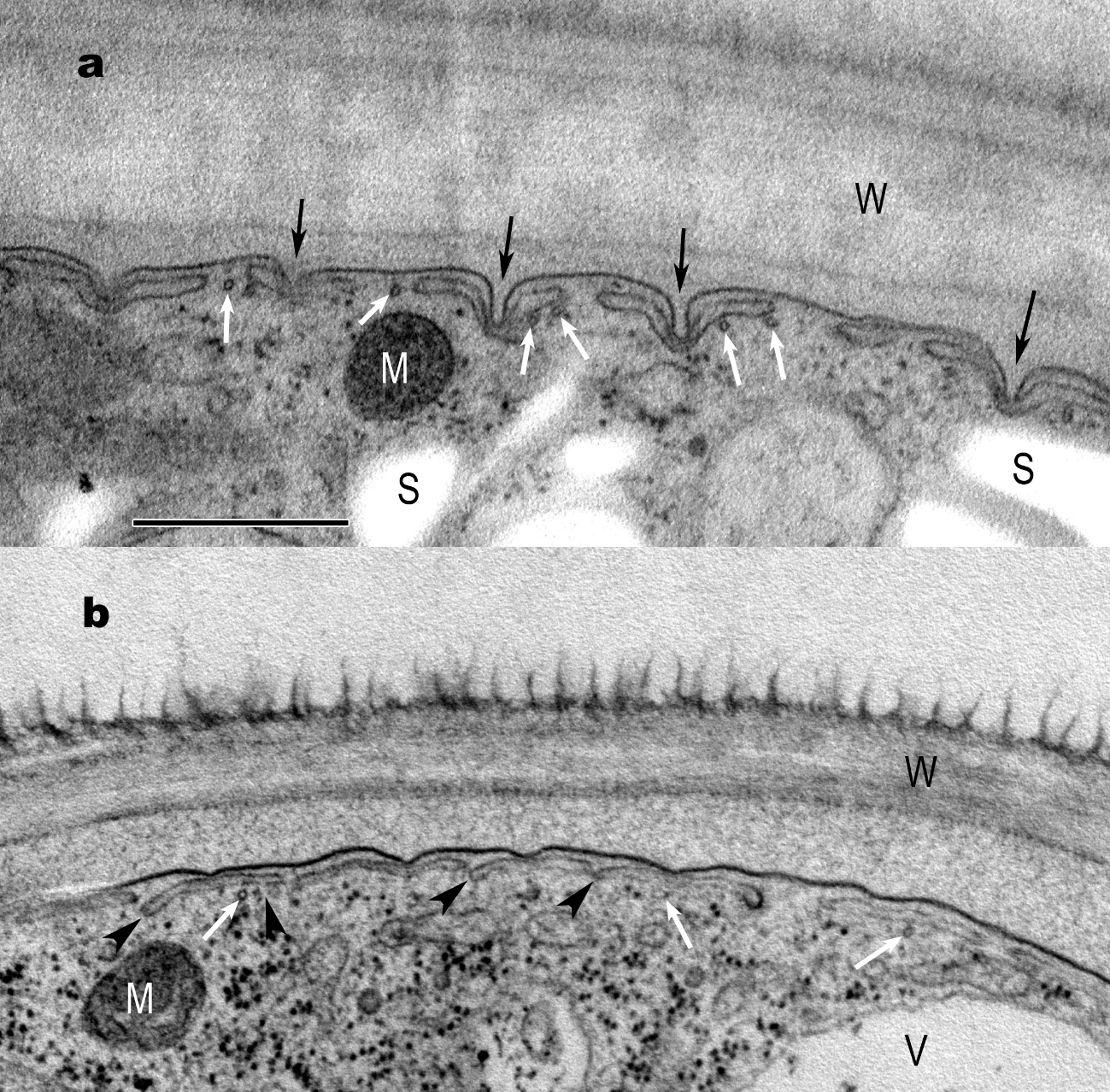


**Supplementary Figure 2 | Ultrathin section transmission electron microscopy of two *Glaucocystis* species.** Shown at the same magnification. M, mitochondrion; S, starch; V, vacuole; W, cell wall; white arrow, microtubule. Scale bar, 500 nm. Note that the cell periphery consists of plasma membrane and underlying flattened vesicles lacking plate-like structure inside. (**a**) “*G.* *geitleri*” SAG 229-1. Note that both plasma membrane and underlying flattened vesicles are deeply grooved (black arrows). (**b**) *G. nostochinearum* SAG 16.98. Note that the plasma membrane lacks deep grooves and that the vesicles are slightly overlapping with one another (arrowheads).

**Supplementary Note 1**

**Results of ultrathin section transmission electron microscopy**

In both species of *Glaucocystis*, a single, continuous plasma membrane enclosed the protoplast, and numerous flattened vesicles closely neighboured the inner surface of the plasma membrane and were distributed throughout the whole protoplast periphery (Supplementary Fig. 2). These vesicles consisted of the outer and inner membranes in section but no plate-like structure was found within the vesicles. The vestigial flagella were observed between the cell wall and the protoplast periphery, positioned always at the equator of the oblate cells. Golgi bodies were distributed within the cytoplasm, and frequently found near the basal bodies of vestigial flagella.

Based on our TEM comparison, the two species exhibited differences in form and arrangement of the flattened vesicles just underneath the plasma membrane (Supplementary Fig. 2). In “*G.* *geitleri*” SAG 229-1, the protoplast showed numerous small depressions at the periphery in section (Supplementary Fig. 2a). Such a depression was shared by the plasma membrane and the centre of the underlying flattened vesicle. At the depression, both plasma membrane and outer and inner membranes of the underlying vesicles were depressed acutely to become an arch-shaped protrusion. The flattened vesicles did not overlap with each other at the protoplast periphery. In *G.* *nostochinearum* SAG 16.98 cells, however, the plasma membrane and the underlying flattened vesicles at the protoplast periphery did not show depressions in section (Supplementary Fig. 2b)*.* The neighbouring flattened vesicles slightly overlapped with one another at the protoplast periphery.

**Supplementary Movie 1 | Ultra-high voltage electron microscopic images of “*Glaucocystis* *geitleri*” SAG 229-1 periphery.** Corresponding to Fig. 2 in the main paper. Note that plasma membrane is grooved deeply.

**Supplementary Movie 2 | Ultra-high voltage electron microscopic tomography and three-dimensional modelling of “*Glaucocystis* *geitleri*” SAG 229-1 periphery.** Corresponding to Fig. 2 in the main paper. Note that each bar-like groove of plasma membrane (magenta) is covered by invagination of flattened vesicle (yellow) associated with microtubules (green) on cytoplasmic side.

**Supplementary Movie 3 | Ultra-high voltage electron microscopic images of *Glaucocystis* *nostochinearum* SAG 16.98 periphery.** Corresponding to Fig. 3 in the main paper. Note that the plasma membrane lacks deep grooves.

**Supplementary Movie 4 | Ultra-high voltage electron microscopic tomography and three-dimensional modelling of *Glaucocystis* *nostochinearum* SAG 16.98 periphery.** Corresponding to Fig. 3 in the main paper. Note that underneath plasma membrane (magenta), flattened vesicles (yellow) slightly overlap each other and associated with microtubules (green) on cytoplasmic side.

**Supplementary Movie 5 | Ultra-high voltage electron microscopic images and tomography of periphery near basal body of “*Glaucocystis* *geitleri*” SAG 229-1**. Corresponding to Fig. 4 in the main paper. Note that the cell periphery in these areas is composed of plasma membrane (asterisks) and ovoid-to-spherical vesicles surrounding basal bodies.

**Supplementary Movie 6 | Ultra-high voltage electron microscopic images and tomography of periphery near basal body of *Glaucocystis nostochinearum* SAG 16.98.** Corresponding to Fig. 4 in the main paper. Note that the cell periphery in these areas is composed of plasma membrane (asterisks) and ovoid-to-spherical vesicles surrounding basal bodies.
